# Self-reported sleep bruxism is associated with vitamin D deficiency and low dietary calcium intake: a case-control study

**DOI:** 10.1186/s12903-020-01349-3

**Published:** 2021-01-07

**Authors:** Mohammad J. Alkhatatbeh, Zainab L. Hmoud, Khalid K. Abdul-Razzak, Esam M. Alem

**Affiliations:** 1 Department of Clinical Pharmacy, Faculty of Pharmacy, Jordan University of Science and Technology, Irbid, 22110 Jordan; 2 Department of Nutrition and Food Technology, Faculty of Agriculture, Jordan University of Science and Technology, Irbid, 22110 Jordan; 3 Department of Prosthodontics, Faculty of Dentistry, Jordan University of Science and Technology, Irbid, 22110 Jordan

**Keywords:** Bruxism, Vitamin D, Calcium, Anxiety, Depression, Headache

## Abstract

**Background:**

Sleep bruxism may result in deleterious effects including loss of tooth enamel, fracture of teeth or restorations, teeth hypersensitivity or pain, and headache. The aim was to study the link between sleep bruxism, low serum vitamin D, low consumption of dietary calcium, psychological symptoms, and frequent headache.

**Methods:**

This case-controlled study included 50 individuals with sleep bruxism and 50 age and gender matched controls. 25-hydroxyvitamin D was measured in serum. Hospital Anxiety and Depression Scale was used to measure anxiety and depression. Data about dietary calcium and frequent headache were self-reported.

**Results:**

Participants with sleep bruxism had lower 25-hydroxyvitamin D and higher scores of anxiety and depression compared to controls (*p* < 0.05). Vitamin D deficiency, abnormal scores of anxiety and depression, low calcium consumption (< 323 mg/day), and frequent headache were reported in higher % of individuals with sleep bruxism compared to controls (*p* < 0.05). Binary logistic regression showed that sleep bruxism was significantly associated with vitamin D deficiency (OR = 6.66, *p* = 0.02), low consumption of dietary calcium (OR = 5.94, *p* = 0.01), and frequent headache (OR = 9.24, *p* < 0.001). Multiple linear regression showed that anxiety was significantly associated with decreased 25-hydroxyvitamin D (*p* = 0.03), increased scores of depression (*p* < 0.001) and female sex (*p* = 0.01). Binary logistic regression also showed that frequent headache was significantly associated with sleep bruxism (OR = 5.51, *p* < 0.01).

**Conclusions:**

Sleep bruxism was associated with vitamin D deficiency and low consumption of calcium and was also associated with increased scores of anxiety and depression. Further investigations should be performed to check if vitamin D and calcium supplementation could relieve sleep bruxism.

## Background

Bruxism is an involuntary frequent jaw muscle movements accompanied by teeth grinding or clenching, which may happen during sleep or while awake [1, 2]. The prevalence rate of awake bruxism is ~ 22–31% while the prevalence rate of sleep bruxism is ~ 9–16% in adults [3–8]. Awake bruxism was found to be predominant in females whereas the prevalence of sleep bruxism had no difference between the two genders [9]. Bruxism is not a life-threatening condition, but it has adverse effects that may influence the quality of sleep and life [10]. For instance, bruxism may increase the risk of teeth sensitivity, fracture, decay, or loss [11]. As well, sleep bruxism may be accompanied by headache as a common symptom that also affects the quality of life [12].

Although bruxism has an uncertain or controversial etiology, many factors could be involved in this disorder including both psychological factors like stress or anxiety and central mechanisms that involve brain neurotransmitters or basal ganglia [13]. A recent study suggested that some psychological elements including personality and stress may contribute to severity and prevalence of the parafunctional behavior [14]. This study found that behaviors such as aggressiveness, neuroticism, perfectionism, and stress characterize people who are more prone to bruxism [14]. This indicates a possible psychological role in the etiology of bruxism.

Previous studies showed that calcium and magnesium deficiencies could be implicated in the development of bruxism through regulating the nervous system and muscular function [15–17]. In addition, there is a suggested epidemiological link between low vitamin D concentrations and various types of sleep problems such as sleep bruxism [18]. So, sleep bruxism could be related to vitamin D deficiency and thus low plasma calcium because sufficient vitamin D levels are required for maintaining calcium homeostasis [19]. Sufficient vitamin D and calcium concentrations are required for regulating both the nervous system and muscular function [20] including the jaw muscle contraction. The possible relationship between vitamin D deficiency and sleep bruxism could also be explained by the vital function of vitamin D and calcium in sustaining mental health as low vitamin D was reported to be associated with psychological symptoms including stress and anxiety [21–23], which themselves could contribute in the development of sleep bruxism [24]. So, the relation between sleep bruxism and psychological issues seems to be the pivotal point of the link between sleep bruxism and low vitamin D.

Previously, we found a significant inverse relationship between anxiety, depression, and total calcium intake, suggesting a connection between low dietary calcium consumption and increased risk of anxiety and depression [25]. Vitamin D administration and increased intake of dietary calcium were effective and safe for the management of muscular pain and accompanying psychological symptoms in individuals with chronic pain, psychiatric outpatients and overactive bladder [21–23]. Thus, it is expected that deficient vitamin D levels and decreased calcium consumption may be involved indirectly in sleep bruxism through their effects on anxiety and depression.

MaCarty and colleagues reported that patients who complained of sleep disruption also showed vitamin D deficiency [26]. Although, there is no evidence to show the exact mechanism that links low vitamin D with sleep disorders. The problem seems to be multifactorial. Some studies showed a relationship between deficient vitamin D levels and non-specific skeletal pain, that may disturb sleep [27]. Other studies demonstrated that vitamin D supplement can relieve pain and improve sleep in subjects who complain of chronic pain [28]. In addition, it was reported that vitamin D has a role in immune regulation, and deficiency in vitamin D can alter immunomodulation and enhance producing cytokines that have roles in sleep [29].

However, these previous studies never investigated the role of nutrition in controlling or diminishing sleep bruxism. Also, no previous study was conducted to investigate the association of low vitamin D levels, low calcium intake, and sleep bruxism. So, we hypothesized that vitamin D levels and intake of dietary calcium are significantly lower in adults with sleep bruxism compared to controls and both vitamin D deficiency and decreased dietary calcium consumption are significantly associated with anxiety and depression, sleep bruxism, and self-reported headache, which is a complication of sleep bruxism. Thus, the main objective from this study was to investigate the relationships between sleep bruxism, low serum vitamin D, low consumption of dietary calcium, psychological symptoms, and frequent headache.

## Methods

### Study design, participants, and sample size

This case-controlled study was performed at Higher Specialty in Dentistry Center and Dental Teaching Clinics of the largest medical teaching university in north of Jordan (latitude is 32^o^ 15 N) between Sep 2019 and Feb 2020. The study involved 50 individuals with sleep bruxism and 50 healthy controls. Cases and controls were matched for both age and gender. Diagnosis of sleep bruxism was made by a specialist of prosthodontics using self-reports and clinical examination. Criteria for the diagnosis were as described in Palinkas et al. study [30]. Individuals were asked to self-report the following symptoms: sounds of teeth grinding while sleeping, temporal headaches, pain in the jaw muscle, muscle fatigue, and locking of the jaw [30]. In addition, visual examinations were done to look for abnormal teeth wears [30]. The 50 cases represent all individuals with sleep bruxism who attended the clinics during the mentioned period with a 100% response rate of participation. The size of the sample was determined according to the mean ± SD of 25-hydroxyvitamin D for healthy adults (17.35 ± 9.81 ng/mL) as reported in our previous study [25]. Based on this standard deviation, we calculated that a sample of 50 was sufficient to obtain a mean difference of ±6.5 between the groups with 90% power, which was considered clinically relevant. The sample size was calculated using the PS: Power and Sample Size Calculation software version 3.0.34 (Vanderbilt Biostatistics, Vanderbilt University Medical Center, Nashville, USA). All participants were checked in the clinic for substance abuse or drug-induced secondary bruxism by checking their medication records and by self-reporting. The controls were healthy persons who visited the clinics for other purposes, university students, or university staff. The controls were apparently healthy with no symptoms of bruxism, musculoskeletal pain, fatigue or sleep problems [22]. Healthy controls did not report any of the symptoms of sleep bruxism that were mentioned above including sounds of teeth grinding while sleeping, temporal headaches, pain in the jaw muscle, muscle fatigue, and locking of the jaw [30] and they did not show any abnormal teen wear on visual examination [30]. Individuals were excluded if they have: diseases that affect vitamin D metabolism such as chronic renal or hepatic diseases, disability problems, diseases that may damage nerves including diabetes and stroke, self-reported migraine headache, history of vitamin D treatment during the previous 2 months [25], or suspected substance abuse or drug-induced bruxism.

Participants were instructed to complete a comprehensive set of guided self-assessment questionnaires. A well-trained research assistant, who was one of the co-authors of this study, was available to answer any question revealed by the participants. The research assistant had received training about the intended meaning of each question in the questionnaire, how to answer these questions, and what was the aim of the study.

### Data collection

Data about participant’s age, marital status, general health status, exercise, complaint of frequent headache, recent vitamin D supplements, and history of chronic diseases were self-reported by the participants. Weight of the body was measured in (kg) after taking off shoes and wearing light clothes and height was measured in (cm). Body Mass Index (BMI) was determined in (kg/m^2^).

### Blood collection and processing

A well-qualified lab technician was responsible for obtaining venous blood samples. Serum was separated by centrifuging the fresh blood at 2000 g for 8 min within 2 h of blood collection. The centrifugation was performed using the high speed centrifuge (centrifuge MR23i, Jouan SA, France).

### 25-hydroxyvitamin D determination

Fresh serum was used to measure 25-hydroxyvitamin D by the electrochemiluminescence immunoassay using the Roche Modular E170 Analyzer (Roche Diagnostics, Basel, Switzerland).

### Determination of daily dietary calcium intake

Daily consumption of calcium from its major dietary resources was determined as we previously described [22, 23, 25, 31, 32]. In brief, participants reported the frequency and amount of their daily intake of various types of dairy products that are found in the market of Jordan (milk, yogurt, cheese, and labaneh) [22, 23, 25, 31]. Consumption of these products was assessed as zero, one, two, or ≥ three dairy servings per day. One serving was defined as: one cup (240 mL) of yogurt or milk (300 mg of calcium), one ounce of cream cheese (20 mg of calcium), one ounce of cheddar cheese (162 mg of calcium), and two tablespoons (two oz) of labaneh (100 mg of calcium) [22, 23, 25, 31]. The sum of daily intake of calcium was computed in mg/day, and the participants were categorized into three groups according to the tertile of their calcium consumption (i.e., < 323 mg/day, 324–600 mg/day, and >  600 mg/day).

### Assessment psychological symptoms

A well-validated Arabic translation of the [33] Hospital Anxiety and Depression Scale (HADS) was used to evaluate psychological symptoms of anxiety and depression [34]. The HADS was translated from English as described in our previous study [25]. The scale is a self-administered and contains seven items used for the assessment of anxiety and other seven items used for the assessment of depression. The score for each question is from zero to three. Thus, the overall scores of both anxiety and depression range from zero to 21. The overall scores were categorized into: normal (0–7), borderline (8–10), and abnormal (11–21).

### Statistical analyses

All statistical analyses were accomplished using the SPSS statistical software version 23 (IBM Corp., Armonk, New York, USA). Frequencies (%) were computed for categorical variables, and medians (25–75th percentiles) were calculated for continuous parameters because they were not normally distributed. Differences between groups in categorical parameters (i.e., sleep bruxism vs. healthy controls and frequent headache vs. no frequent headache) were examined using chi-squared test. Differences between groups in continuous parameters (i.e., sleep bruxism vs. healthy controls and frequent headache vs. no frequent headache) were assessed by Mann-Whitney U test. Correlations between continuous parameters were analyzed using Spearman’s correlation test. Binary logistic regression test was considered to find associations between sleep bruxism and other independent variables including self-reported frequent headache, anxiety, depression, dietary calcium intake, and 25-hydroxyvitamin D. In addition, binary logistic regression test was considered to find associations between self-reported frequent headache and other independent variables including sleep bruxism, anxiety, and depression. Multiple linear regressions were considered to find the associations between anxiety, depression, and other independent variables including gender, marital status, occupation, and serum 25-hydroxyvitamin D. All statistical tests were 2-tailed, and only *p* values below 0.05 were considered significant.

## Results

### Characteristics of participants

The sample consisted of 50 individuals with sleep bruxism and 50 age and gender matched healthy controls. 32% (*n* = 32) of the participants were males and 68% (*n* = 68) were females. Their median age and BMI (25th–75th percentile) were 35 (25–45.75) years and 25.37 (22.28–28.33) kg/m^2^, respectively. Other general characteristics are shown in Table 1.

**Table 1:** Differences in variables between participants with sleep bruxism and controls

Variable	Total ***n*** = 100	Participants with sleep bruxism (***n*** = 50)	Healthy controls (***n*** = 50)	***P***-value^a^
Age (Years)	35 (25–45.75)	35.0 (25.0–45.50)	35.50 (25.75–46.25)	0.96
Gender				
Male	32 (32)	16 (32)	16 (32)	1.00
Female	68 (68)	34 (68)	34 (68)	
Marital status				
Single	46 (46)	23 (46)	23 (46)	1.00
Married	54 (54)	27 (54)	27 (54)	
BMI (kg/m^2^)	25.37 (22.28–28.33)	24.97 (22.45–27.85)	26.15 (21.11–29.01)	0.94
General health status				
Excellent	27 (27)	13 (26)	14 (28)	0.15
Very good	51 (51)	22 (44)	29 (58)	
Good	22 (22)	15 (30)	7 (14)	
Regular exercise				
Yes	67 (67)	37 (74)	30 (60)	0.20
No	33 (33)	13 (26)	20 (40)	
Self-reported frequent headache				
Yes	54 (54)	38 (76)	16 (32)	**< 0.001**
No	46 (46)	12 (24)	34 (68)	
HADS-anxiety score (0–21)	6 (3–10)	7 (5–11)	5 (2–7)	**< 0.001**
Normal (0–7)	70 (70)	27 (54)	43 (86)	**< 0.01**
Borderline (8–10)	11 (11)	9 (18)	2 (4)	
Abnormal (11–21)	19 (19)	14 (28)	5 (10)	
HADS-depression score (0–21)	6 (3–9)	8 (4–11)	5 (2–7)	**< 0.001**
Normal (0–7)	65 (65)	24 (48)	41 (82)	**< 0.01**
Borderline (8–10)	20 (20)	13 (26)	7 (14)	
Abnormal (11–21)	15 (15)	13 (26)	2 (4)	
Dietary calcium intake (mg/day)	457.49 (303.10–716.95)	464.50 (322.0–620.75)	400.02 (286.29–752.42)	0.96
< 323 mg/day	33 (33)	13 (26)	20 (40)	**0.01**
324–600 mg/day	33 (33)	24 (48)	9 (18)	
> 600 mg/day	34 (34)	13 (26)	21 (42)	
Serum 25-hydroxyvitamin D ng/mL	21 (13–32)	17.5 (11–25.25)	29 (15.75–36.25)	**< 0.01**
Sufficient (≥ 30 ng/mL)	32 (32)	9 (18)	23 (46)	**0.01**
Insufficient (20–30 ng/mL)	21 (21)	11 (22)	10 (20)	
Deficient (< 20 ng/mL)	47 (47)	30 (60)	17 (34)	

^a^Mann-Whitney U test, Chi-square test as appropriate (*p* values were considered significant at < 0.05 are shown in bold). *HADS* Hospital Anxiety and Depression Scale, *BMI* Body Mass Index

### Vitamin D status among study participants and association with sleep bruxism

Vitamin D deficiency was reported in 47 (47%) of all participants. Chi-squared test (Table 1) has shown that there was a significant association between vitamin D deficiency and sleep bruxism (*p* = 0.01), as 60% of the participants with sleep bruxism had deficient vitamin D levels compared to 34% of the controls. In addition, Table 1 showed that participants with sleep bruxism had significantly lower serum 25-hydroxyvitamin D levels compared to the controls (*p* < 0.01).

### Status of daily dietary calcium intake among study participants and association with sleep bruxism

Most of the study participants (90%) reported daily dietary calcium intake that is less than the Recommended Daily Intake of calcium (< 1000 mg/day) [35]. However, when we divided the participants according to the tertile of their daily dietary calcium intake, Chi-squared test (Table 1) showed that sleep bruxism was significantly associated with lower daily dietary calcium intake (*p* = 0.01) as 26% of the participants with sleep bruxism had daily dietary calcium intake > 600 mg/day compared to 42% of the controls.

### Status of anxiety and depression and association with sleep bruxism

Abnormal (clinical) and borderline HADS-anxiety scores were reported in 19 and 11% of the participants, respectively. As well, abnormal and borderline HADS-depression scores were reported in 15 and 20% of the participants, respectively. Chi-squared test (Table 1) showed that sleep bruxism was significantly associated with abnormal HADS-anxiety scores (*p* < 0.01) as 28% of participants with sleep bruxism had abnormal HADS-anxiety scores compared to 10% of the controls. Similarly, sleep bruxism was significantly associated with abnormal HADS-depression scores (p < 0.01) as 26% of participants with sleep bruxism had abnormal HADS-anxiety scores compared to 4% of the controls. Additionally, Table 1 showed that participants with sleep bruxism had significantly higher HADS-anxiety scores and HADS- depression scores compared to the controls (*p* < 0.001).

### Self-reported frequent headache and association with sleep bruxism

Frequent headache was reported by 54% (*n* = 54) of all participants. Chi-squared test showed that sleep bruxism was significantly associated with self-reported frequent headache (*p* < 0.001) as 76% (*n* = 38) of participants with sleep bruxism were complaining of frequent headache compared to 32% (*n* = 16) of the controls.

### Correlation between 25-hydroxyvitamin D, dietary calcium intake and other variables

As shown in Table 2, there was significant inverse correlation between HADS-anxiety scores and 25-hydroxyvitamin D concentrations (r = − 0.25, *p* = 0.01). Also, there was significant correlation between HADS-anxiety and depression scores (r = 0.64, *p* < 0.001) and between age and BMI (r = 0.49, *p* < 0.001).

**Table 2:** Correlation between 25-hydroxyvitamin, daily dietary calcium intake and other variables in all participants

	BMI (kg/m^**2**^)	HADS-anxiety score	HADS- depression score	Daily dietary calcium intake (mg/day)	Serum 25-hydroxyvitamin D (ng/mL)
Age (Years)	r = 0.49 *p* **< 0.001**	r = −0.15 *p* = 0.14	r = 0.08 *p* = 0.46	r = 0.09 *p* = 0.40	r = 0.16 *p* = 0.11
BMI (kg/m^2^)	–	r = −0.12 *p* = 0.23	r = 0.04 *p* = 0.73	r = 0.06 *p* = 0.59	r < 0.01 *p* = 0.98
HADS-anxiety score	–	–	r = 0.64 *p* **< 0.001**	r = 0.05 *p* = 0.66	r = −0.25 *p* = **0.01**
HADS- depression score	–	–	–	r = −0.05 *p* = 0.64	r = −0.13 *p* = 0.21
Daily dietary calcium intake (mg/day)	–	–	–	–	r = 0.17 *p* = 0.09

Spearman correlation analysis (*p*-values were 2-tailed and considered significant at < 0.05 are shown in bold). r, Spearman’s correlation coefficient; *HADS* Hospital Anxiety and Depression Scale, *BMI* Body Mass Index

### Association of sleep bruxism with other variables

Binary logistic regression analysis (Table 3) showed that sleep bruxism was significantly associated with deficient vitamin D levels (OR = 6.66, *p* = 0.02), insufficient vitamin D levels (OR = 11.69, *p* < 0.01), low daily dietary calcium intake (< 323 mg/day, OR = 5.94, *p* = 0.01), and self-reported frequent headache (OR = 9.24, *p* < 0.001).

**Table 3:** Association of sleep bruxism with vitamin D and other variable

Variable	Value	B (SE)	Odds ratio	Confidence interval	***P***-value^a^
Constant	–	−3.95 (0.94)	–	–	< 0.001
Self-reported frequent headache	Yes	2.22 (0.62)	9.24	2.74–31.21	**< 0.001**
	No (reference)	–	–	–	
HADS- anxiety score	Abnormal	1.44 (0.83)	4.20	0.83–21.22	0.08
	Borderline	2.05 (1.12)	7.74	0.86–69.29	0.07
	Normal (reference)	–	–	–	0.07
HADS- depression score	Abnormal	1.60 (1.09)	4.97	0.59–42.11	0.14
	Borderline	0.48 (0.70)	1.62	0.41–6.35	0.49
	Normal (reference)	–	–	–	0.31
Daily dietary calcium intake (mg/day)	< 323 mg/day	1.78 (0.71)	5.94	1.47–23.99	**0.01**
	324–600 mg/day	−0.38 (0.70)	0.68	0.17–2.68	0.59
	> 600 mg/day (reference)	–	–	–	0.01
Serum 25-hydroxyvitamin D (ng/mL)	Deficient	1.90 (0.83)	6.66	1.31–33.85	**0.02**
	Insufficient	2.46 (0.77)	11.69	2.57–53.18	**< 0.01**
	Sufficient (reference)	–	–	–	0.01

^a^ Binary logistic regression (dependent variable: sleep bruxism versus controls), *p*-values < 0.05 was considered statistically significant are shown in bold. B: coefficient (intercept); *SE* Standard error, *HADS* Hospital anxiety and depression score

### Association of anxiety and depression with other variables

As shown in Table 4, multiple linear regression model revealed that increased HADS-anxiety score was significantly associated with female gender (*p* = 0.01), increased HADS-depression score (*p* < 0.001), and decreased 25-hydroxyvitamin D concentration (*p* = 0.03). Increased HADS-depression score was significantly associated with increased HADS-anxiety score (*p* < 0.001) but not with 25-hydroxyvitamin D concentration (*p* = 0.73).

**Table 4:** Association of anxiety and depression with other variables

Variable	R^**2**^	ANOVA	Model	B	β	***P***-value^a^
HADS- anxiety score	0.53	F = 20.82, *p*-value < 0.001	Constant	0.15	–	0.95
			Gender	2.31	0.22	**0.01**
			Marital status	0.05	0.01	0.95
			Occupation	−0.27	−0.11	0.19
			HADS- depression score	0.76	0.63	**< 0.001**
			Serum 25-hydroxyvitamin D	−0.06	−0.16	**0.03**
HADS- depression score	0.46	F = 16.20, *p*-value < 0.001	Constant	3.96	–	< 0.05
			Gender	−1.44	−0.17	0.06
			Marital status	0.19	0.02	0.79
			Occupation	0.13	0.06	0.49
			HADS- anxiety score	0.59	0.71	**< 0.001**
			Serum 25-hydroxyvitamin D	0.01	0.03	0.73

^a^ Multiple linear regression analysis (*p*-values < 0.05 were considered statistically significant are shown in bold); R^2^, squared coefficient of determination; *B* unstandardized coefficient, *β* standardized coefficient, *F* F-statistic, *HADS* Hospital Anxiety and Depression Score

### Association of frequent headache with other variables and differences according to headache status

As shown in Table 5, participants with self-reported frequent headache were having significantly higher HADS-anxiety (*p* < 0.01) scores and HADS- depression scores (*p* < 0.001) compared to participants who did not report frequent headache. As well, Chi-squared test showed significant association between self-reported frequent headache and abnormal HADS-depression status (*p* = 0.01) as abnormal HADS-depression scores were reported in 22.2% of participants with frequent headache compared to 6.5% of participants who did not report having frequent headache. Further binary logistic regression analysis (Table 6) showed that frequent headache was significantly associated with sleep bruxism (OR = 5.51, *p* < 0.01).

**Table 5:** Differences in variables according to self-reported frequent headache status

Variable	Participants with frequent headache (***n*** = 54)	Participants with no frequent headache (***n*** = 46)	***P***-value^a^
Age (Years)	34 (23.75–43.5)	36.5 (27–47.25)	0.12
Gender			
Male	13 (24.1)	19 (41.3)	0.07
Female	41 (75.9)	27 (58.7)	
Marital status			0.39
Single	27 (50)	19 (41.3)	
Married	27 (50)	27 (58.7)	
BMI (kg/m^2^)	24.85 (22.12–27.71)	26.15 (22.29–29.23)	0.41
General health status			
Excellent	12 (22.2)	15 (32.6)	0.50
Very good	29 (53.7)	22 (47.8)	
Good	13 (24.1)	9 (19.6)	
Regular exercise			0.73
Yes	37 (68.5)	30 (65.2)	
No	17 (31.5)	16 (34.8)	
HADS-anxiety score (0–21)	7 (4.75–10)	5 (1–7)	**< 0.01**
Normal (0–7)	34 (63)	36 (78.3)	0.22
Borderline (8–10)	8 (14.8)	3 (6.5)	
Abnormal (11–21)	12 (22.2)	7 (15.2)	
HADS-depression score (0–21)	7 (4.75–10)	4 (2–7)	**< 0.001**
Normal (0–7)	28 (51.9)	37 (80.4)	**0.01**
Borderline (8–10)	14 (25.9)	6 (13)	
Abnormal (11–21)	12 (22.2)	3 (6.5)	
Dietary calcium intake (mg/day)	405 (303.70–638.75)	469.40 (298.75–752.84)	0.61
< 323 mg/day	18 (33.3)	15 (32.6)	0.28
324–600 mg/day	21 (38.9)	12 (26.1)	
> 600 mg/day	15 (27.8)	19 (41.3)	
Serum 25-hydroxyvitamin D (ng/mL)	21 (12.75–31.25)	21 (13.75–33.25)	0.61
Sufficient (≥ 30 ng/mL)	16 (29.6)	16 (34.8)	0.85
Insufficient (20–30 ng/mL)	12 (22.2)	9 (19.6)	
Deficient (< 20 ng/mL)	26 (48.1)	21 (45.7)	

^a^ Mann-Whitney U test or Chi-square test as appropriate (*p* values were considered significant at < 0.05 are shown in bold). *HADS* Hospital Anxiety and Depression Scale, *BMI* Body Mass Index

**Table 6:** Association of self-reported frequent headache with other variables

Variable	Value	B (SE)	Odds ratio	Confidence interval	***P***-value^a^
Constant	–	−0.81 (0.44)	–	–	0.06
Sleep bruxism	Yes	1.71 (0.49)	5.51	2.10–14.45	**< 0.01**
	No (reference)	–	–	–	
HADS- anxiety score	Abnormal	0.51 (0.68)	1.66	0.44–6.27	0.45
	Borderline	−0.10 (0.84)	0.90	0.17–4.68	0.90
	Normal (reference)	–	–	–	0.72
HADS- depression score	Abnormal	−1.11 (0.79)	0.33	0.07–1.53	0.16
	Borderline	−0.94 (0.64)	0.39	0.11–1.36	0.14
	Normal (reference)	–	–	–	0.19

^a^Binary logistic regression (dependent variable: sleep bruxism versus controls), *p*-values < 0.05 was considered statistically significant are shown in bold. B: coefficient (intercept); *SE* Standard Error, *HADS* Hospital Anxiety and Depression Score

## Discussion

This study had investigated the relationship between serum vitamin D, calcium intake, anxiety, depression, and self-reported frequent headache in participants with sleep bruxism compared to healthy controls. Our hypothesis was that sleep bruxism is associated with vitamin D deficiency, low dietary calcium intake, anxiety, depression, and headache, which could be a complication of sleep bruxism itself [12]. This hypothesis was based on our previous studies that found an association between vitamin D deficiency, low dietary calcium consumption, anxiety, depression, and musculoskeletal function [21–23, 25]. Since sleep bruxism is considered as a parafunctional activity that is associated with repetitive jaw muscle hyperactivity [36], similar association between sleep bruxism, vitamin D deficiency, and low dietary calcium consumption [21–23, 25] was expected. Likewise, frequent headache that may result from musculoskeletal pain could also be linked to vitamin D deficiency [37].

Results of the current study showed that scores of anxiety and depression were significantly higher and 25-hydroxyvitamin D was significantly lower in participants with sleep bruxism compared to the controls. As well, participants with sleep bruxism were complaining of headache and having lower levels of daily dietary calcium intake much more than controls. These findings suggest that anxiety, depression, vitamin D deficiency, low level of dietary calcium intake, and frequent headache could be involved in the development of sleep bruxism. So, Chi-squared testing was conducted to check association between sleep bruxism and categories of anxiety, depression, calcium intake, vitamin D, and headache. Results revealed significant association between sleep bruxism and vitamin D deficiency, low dietary calcium intake, abnormal HADS-anxiety and depression categories, and frequent headache. However, further regression analysis revealed that sleep bruxism was significantly associated with vitamin D deficiency and insufficiency, low dietary calcium, and frequent headache but it was not associated with anxiety or depression.

Our results regarding the relationship between sleep bruxism and the psychological symptoms were consistent with the results reported in Gungormus Z et al. [38] study, in which anxiety and depression scores were significantly higher in individuals with sleep bruxism compared to individuals with no sleep bruxism. Similarly, Ahlberg J et al. [24] study showed that subjects with more frequent sleep bruxism were having more severe anxiety. The relationship between sleep bruxism and the psychological symptoms could be explained by their effect on the musculoskeletal function as sleep bruxism is accompanied by repetitive jaw muscle hyperactivity [36]. Muscle tension is considered as a common anxiety symptom that increases strain on the muscle and with time it may end with musculoskeletal symptoms such as pain [39, 40].

The novel finding of our study was the significant association between sleep bruxism and vitamin D deficiency. To the best of our knowledge, there was no previous study that addressed this relationship. This association can be justified by the role of vitamin D in maintaining mental health as vitamin D deficiency was reported to be associated with psychological symptoms including anxiety, which itself may lead to sleep bruxism [21–23]. Vitamin D has a neuroprotective effect that is associated with its effect on the production of neurotrophin, synthesis of neuromediators, regulation of intracellular calcium levels, and reduction of oxidative damage to the central nervous system [41]. So, low vitamin D levels may dysregulate calcium homeostasis in the brain, which may influence neuron excitability [41] and lead to increased risk of psychiatric problems such as dementia, schizophrenia, anxiety, and depression [42]. Our findings also support this explanation as vitamin D deficiency was significantly associated with anxiety.

Additionally, the traditional function of vitamin D is maintaining calcium homeostasis and thus low vitamin D leads to low serum calcium levels [19]. Hypocalcemia has direct effects on the neuromuscular function that may cause muscle spasm and cramps [43]. Again, because sleep bruxism involves repetitive jaw muscle hyperactivity, vitamin D deficiency and its associated hypocalcemia may be implicated in the development of sleep bruxism through their effect on the neuromuscular function. This explanation is supported by the possible treatment of sleep bruxism through replacement of magnesium and calcium deficiencies [44]. In addition, the current study showed that low calcium intake was significantly associated with sleep bruxism. This also supports that low serum calcium levels could be involved in the development of sleep bruxism as discussed above.

Another important finding of our study was the relationship between frequent headache and sleep bruxism. Although participants with headache had significantly higher scores of anxiety and depression compared to participants with no headache, both anxiety and depression were not significantly associated with headache. Headache was only significantly associated with sleep bruxism. This suggests that sleep bruxism may result in frequent headache. This result was confirmed by Das S et al. [45] study, which had reported that sleep-related bruxism may present with headache. The association between sleep bruxism and headache could be due to the excessive clenching or grinding that affect the surrounding muscles leading to prolonged muscle contraction, tension, and headache [46].

Taken together, this study has reported a novel association between sleep bruxism, vitamin D deficiency, low dietary calcium intake, psychological symptoms, and self-reported frequent headache. The case-control study design is considered as a main strength of the study because all associations were based on comparisons with age and gender matched healthy individuals who were not complaining of sleep bruxism. However, the current study has some limitations that may prevent the inference of certain conclusions that can be generalized. Our small sample size was limited by the small number of bruxers who attended the clinic. The study was conducted at clinics that serve the university population where the researchers of this study belong to. So, there was a possibility that some participants might be influenced directly or indirectly by the researchers. Because the university population is relatively large and the clinics also serve the local community of the university area, we believe that this influence is minimal. Diagnosis of sleep bruxism was not confirmed by polysomnography because it is expensive, time consuming and not available in the place where the study was conducted. Polysomnography, is the standard method for diagnosing sleep bruxism and it has little agreement with the diagnosis that is based on self-reports and clinical examination [47]. However, there is no perfect method to diagnose sleep bruxism and diagnosis by self-reports and clinical examination in combination can be used to diagnose sleep bruxism [47]. Other study parameters including the dietary calcium consumption, anxiety, depression, and headache were assessed by self-reporting. Even though, the assessment of dietary calcium consumption and both anxiety and depression were assessed by well-validated questionnaires that were approved for clinical research purposes and used by many researchers as described in the Methods section. Unfortunately, we were unable to apply any classification method to determine the type of self-reported frequent headache among our participants. So, our results cannot be applied to a specific type of headache. Regardless of these limitations, we still believe that our results are novel and reported here for the first time. We also believe that this study will motivate others to do relevant investigations to check if there is a cause-effect relationship between sleep bruxism and both vitamin D deficiency and low calcium. In addition, further studies are essential to check if correcting deficient vitamin D levels, increasing dietary calcium intake, and treatment of anxiety and depression may relieve symptoms of sleep bruxism. A future study with a similar design that includes external participants is also recommended to ensure that there will be no influence on participants from the researchers of the same institution.

## Conclusions

This study has reported a novel association between sleep bruxism, vitamin D deficiency, low dietary calcium intake, anxiety, depression, and frequent headache. Vitamin D deficiency, abnormal scores of anxiety and depression, low dietary calcium, and frequent headache were reported in higher % of participants with sleep bruxism compared to controls. Sleep bruxism was significantly associated with vitamin D deficiency and low dietary calcium intake and is also associated with increased anxiety and depression scores. Further investigations are needed to check if vitamin D and calcium supplementation can improve sleep bruxism.

## Data Availability

The datasets used and/or analysed during the current study are available from the corresponding author on reasonable request.

## Abbreviations

HADS: Hospital Anxiety and Depression Scale
BMI: Body mass index
OR: Odds ratio
B: Coefficient
SE: Standard error
R^2^: Squared coefficient of determination
Β: Standardized coefficient
F: F-statistic
